# Long non‐coding RNA 00312 regulated by HOXA5 inhibits tumour proliferation and promotes apoptosis in Non‐small cell lung cancer

**DOI:** 10.1111/jcmm.13142

**Published:** 2017-03-24

**Authors:** Qingqing Zhu, Tangfeng Lv, Ying Wu, Xuefei Shi, Hongbing Liu, Yong Song

**Affiliations:** ^1^ Department of Respiratory Medicine Jinling Hospital Nanjing University School of Medicine Nanjing China; ^2^ Nanjing University Institute of Respiratory Medicine Nanjing China; ^3^ Department of Respiratory Medicine Zhongda Hospital Southeast University School of Medicine Nanjing China; ^4^ Department of Respiratory Medicine Huzhou Central Hospital Huzhou China

**Keywords:** NCSLC, lncRNA, linc00312, HOXA5, proliferation, apoptosis

## Abstract

Non‐small cell lung cancer (NSCLC) is the most prevalent type of lung cancer. The abnormal expression of many long non‐coding RNAs (lncRNAs) has been reported involved in the progression of various tumours, which can be used as diagnostic indicators or antitumour targets. Here, we found that the long non‐coding RNA 00312 was down‐regulated in paired NSCLC tissues and correlated with poor clinical outcome; decreased linc00312 expression in NSCLC was associated with larger and later stage tumours. Functional experiments showed that linc00312 could inhibit cell proliferation and promote apoptosis *in vitro* and *in vivo*. Furthermore, we found that HOXA5 could bind in the promoter of linc00312 and up‐regulated the expression of it. Moreover, linc00312 was down‐regulated in the plasma of NSCLC patients compared with that of healthy volunteers or other pulmonary diseases patients. Taken together, our findings indicated that linc00312 could be a novel diagnosis biomarker and a promising therapeutic target for NSCLC.

## Introduction

Lung cancer is the most frequently diagnosed cancer and remains the number one cancer‐related cause of death in China and worldwide, which is one of the heaviest cancer burdens 1, 2. The majority (~80%) of lung cancer cases are NSCLC comprising two major subtypes, adenocarcinoma (ADC) and squamous cell carcinoma (SCC) 3. Even though we have made great progress in the therapies of lung cancer, the overall 5‐year survival rate of the disease is less than 15% 4. Thus, it remains a priority of exploring the early detection and treatment targets for NSCLC. More importantly, it is urged to blow away the myths of the mechanism of lung cancer.

LncRNAs are a class of transcribed RNA molecules which have a length >200 nucleotides that may not encode proteins. Accumulating studies have demonstrated that lncRNAs are emerging as key regulators involved in various biological processes 5, 6, 7 and a broad range of diseases 8, 9. According to their location with respect to the nearest protein‐coding transcripts, lncRNAs can be categorized as genic or intergenic lncRNAs 10. Long intergenic non‐coding RNAs (lincRNAs) do not overlap exons of either protein‐coding or other types of genes 11. A growing number of researches have shown that lncRNAs could regulate the tumorigenesis and development of tumours, which indicates that lncRNAs may serve as novel therapeutic targets in cancers 12. To date, there are a lot of lncRNAs have been shown in the progression of lung cancer (Table S1, data from the Lnc2cancer database). However, compared with the vast majority of lncRNAs in the genome, the function of lncRNAs in NSCLC is only about to be unravelled. Thus, the increasing mechanistic understanding may provide useful disease information with high predictive power and potential treatment targets. We previously showed that GAS5 (growth arrest‐specific transcript 5) was down‐regulated in NSCLC and increased tumour cell growth arrest and induced apoptosis 13. In addition, we also discovered two up‐regulated lncRNAs, HNF1A‐AS1 and linc00673. Linc00673 could exert its oncogenic function *via* binding LSD1 and then inhibiting neurocalcin delta (NCALD) expression in NSCLC 14. HNF1A‐AS1 might bind to DNMT1 and regulated related gene expression resulting in proliferation and metastasis of ADC 15. Nevertheless, our understanding of the mechanisms of lncRNAs in NSCLC still largely remains elusive. In this regard, further studies are needed.

Long non‐coding RNA 00312(linc00312)is a lincRNA located on 3p25.3. It was reported to be negatively correlated with tumour size but positively correlated with lymph node metastasis in nasopharyngeal carcinoma 16. In addition, a recent study showed that linc00312 expression was lower in bladder cancer tissues and it could inhibit bladder cancer cell invasion and metastasis through mediating miR‐197‐3p 17. Hui Yu *et al*. performed an integrative analysis of two NSCLC microarray data sets (GSE19188 and GSE18842) and found that linc00312 was one of the most down‐regulated lncRNA in NSCLC tumours 18, which indicates that linc00312 may be associated with tumorigenesis or histological differentiation in NSCLC. In this regard, it is essential to further explore the function of linc00312 in NSCLC.

Homeobox genes comprise a family of regulatory genes, which contain a common homeobox domain and act as transcription factors. Homeobox A5 (HOXA5) is one of the homeobox genes and was reported down‐regulated in NSCLC. In addition, HOXA5 could inhibit metastasis *via* binding to the promoters of cytoskeleton‐related genes and down‐regulating their expression. 19. The high‐expressing HOXA5 was associated with prolonged survival in NSCLC and suppresses cell proliferation by regulating p21 20. However, little is known about HOXA5 and lncRNAs in NSCLC.

In this study, we presented for the first time a detailed analysis of linc00312 in NSCLC. We found that linc00312 was obviously down‐regulated in paired NSCLC tissues and patients’ plasma. Linc00312 overexpression resulted in a decrease in the proliferation of lung tumour both *in vivo* and *vitro*. Also, theHOXA5/linc00312 axis might be important in this progress.

## Materials and methods

### Gene expression data sets

Three human microarray data sets including GSE19804, GSE10072 and GSE66654 were obtained from public Gene Expression Omnibus (GEO) database (http://www.ncbi.nlm.nih.gov/geo) and normalized using Robust Multichip Average (RMA). The Cancer Genome Atlas (TCGA) data were used from the Atlas of ncRNA in cancer (TANRIC:http://ibl.mdanderson.org/tanric/_design/basic/query.html). Two‐class paired or unpaired analyses were used according to experimental design.

### Patients, tissue samples and blood samples

We obtained paired NSCLC and adjacent normal lung tissues from 100 patients who underwent primary surgical resection in the Department of Thoracic Surgery of Jinling Hospital from February 2014 to May 2016. No patients had received any anticancer treatments before operation. All tissue samples collected following surgical removal were immersed in RNA Later stabilization solution (Qiagen, Hilden, Germany) and were immediately frozen in liquid nitrogen. The NSCLC or inflammatory patient blood was obtained from the Department of Respiratory Medicine of Jinling Hospital, and the healthy control blood was obtained from people who received medical examination in Jinling Hospital. Plasma samples were collected before any anticancer treatments were given. All NSCLC patients were clearly diagnosed based on histopathology or biopsy analysis. Our study protocol was approved by the Institutional Review Board, Nanjing University of Medicine. All of the participants signed an informed consent form, and the research was carried out according to the World Medical Association Declaration of Helsinki.

### Cell culture

The ADC cell lines (A549, SPC‐A1, H1299, H1975, PC9) and SCC cell lines (H1703, H520 and SK‐MES) were obtained from the Institute of Biochemistry and Cell Biology of the Chinese Academy of Sciences (Shanghai, China). A549, H1299, H1703, H520, H1975, SK‐MES and SPC‐A1 cells were maintained in RPMI Medium 1640 basic media (GIBCOBRL, Invitrogen, Carlsbad, CA, USA). PC9, 16HBE cells were grown in DMEM (DGIBCOBRL, Invitrogen, Carlsbad, CA, USA). The 1640 or DMEM media were supplemented with 10% foetal bovine plasma (FBS, HyClone, Camarillo, CA, USA). All the cells were cultured in a humidified incubator at 37°C with 5% CO_2_.

### RNA isolation, complementary DNA (cDNA) synthesis and quantitative reverse transcriptase polymerase chain reaction (qRT‐PCR) analyses

Total RNA was extracted from NSCLC cells or tissues using TRIZOL Reagent (Invitrogen, Carlsbad, CA, USA) following the manufacturer's protocol. Total RNA was extracted from plasma using mirVana^TM^ PARIS kit (Ambion, Austin, TX, USA) according to the manufacturer's protocol. The cDNA was synthesized using the Primer‐Script™ One Step RT‐PCR Kit (TaKaRa, Dalian, China). Quantitative real‐time PCR was carried out on Quant studio 3 (Applied Biosystems, Foster city, CA, USA) using a SYBR Premix Ex Taq II kit (TaKaRa) according to the manufacturer's instructions. Glyceraldehyde‐3‐phosphate dehydrogenase (GAPDH) was used for normalization. The relative expression fold change of mRNAs was calculated by the 2^−ΔΔCt^ method. The calculation of delta Ct value was performed as follows: ΔCt(target) = Ct (target) − Ct (GAPDH). The primer sequences used in this study were listed in Supplement Table S2.

### Cell transfection

siRNA was transfected into cells using Lipofectamine 2000 (Invitrogen, Shanghai, China), according to the manufacturers’ instructions. Sequences of siRNAs are described in Supplementary Table S2. Plasmid vectors were transfected into cells using X‐treme GENE HP DNA transfection reagent (Roche, Basel, Switzerland) according to the manufacturer’ instructions. Linc00312 overexpression plasmid was created based on the NCBI Reference Sequence, NR_024065.2. The HOXA5 overexpression plasmid was synthesized according to NCBI Reference Sequence. pcDNA3.1 vector was used as plasmid vector and empty control (Invitrogen, Shanghai, China). After 48‐h transfection, cells were used for further experiments..

### Isolation of cytoplasmic and nuclear RNA

Cytoplasmic and nuclear RNA were separated and purified using the PARIS Kit (Life Technologies, Carlsbad, CA, USA) following the manufacturers’ instructions.

### Cell proliferation assays

Cell proliferation ability was examined using a Cell Proliferation Reagent Kit I (MTT) (Roche) and EDU assay kit (Ribobio, Guangzhou, China). Colony formation assays were performed to monitor NSCLC cells cloning capability.

### Cell apoptosis assays

A549, SPC‐A1 and PC9 cells transfected with si‐linc00312/si‐NC or pcDNA3.1‐linc00312/ empty vector were harvested 48 hrs with trypsinization. The cells were diluted with the binding buffer and staining with FITC‐Annexin V (AV) and propidium iodide (PI) and then analysed with a flow cytometry (FACScan^®^; BD Biosciences, San Jose, CA, USA) equipped with the CellQuest software (BD Biosciences).

### Xenograft mouse model

Male and 4‐week‐old athymic BALB/c nude mice were used for the tumour formation assay. SPCA1 cells (1 × 10^6^) stably expressing pcDNA3.1‐linc00312 or an empty vector were subcutaneously injected into either side of the flank area of the mice (*n* = 6 mice per group). The tumour volume was measured every 4 days. The tumours were removed from all mice after 16 days. Animal care and experimental protocol were approved by the Model Animal Research Center of Jingling Hospital and conducted according to Institutional Animal Care and User Guidelines.

### Immunohistochemistry (IHC)

Tumour specimens from nude mice were fixed in 4% paraformaldehyde and then embedded in paraffin. Sections were used for the analysis of Ki67 (1:200; Cell Signal Technology, Danvers, MA, USA), hematein–eosin (HE) and tunnel with dUTP kits (Roche).

### Chromatin immunoprecipitation (ChIP) assays

We used the EZ‐Magna ChIP kit (Millipore, Billerica, MA, USA) to carry out the assays according to the protocol. High‐quality formaldehyde was used to incubate the cells to generate DNA‐protein cross‐links. Cell lysates were then sonicated to generate chromatin fragments of 200–300 bp and immunoprecipitated with HOXA5 (Abcam, Cambridge, MA, USA) and with IgG (Millipore) as negative control or anti‐acetyl histone H3 as positive control (Millipore). Precipitated chromatin DNA was recovered and analysed by qRT‐PCR. The specific primers were listed in Supplement Table S2.

### Statistical analysis

All statistical analyses in this study were performed using SPSS 22.0 software (IBM, Chicago, IL, USA), and *P* < 0.05 was considered to be significant. Data are presented as mean ± standard deviation (S.D.). Student's *t*‐test was used to compare differences. Pearson's correlation analysis was applied to analyse the correlation between linc00312 and HOXA5 in tissues.

## Results

### Expression profiles of linc00312 in NSCLC GEO data sets

**Figure 1:**
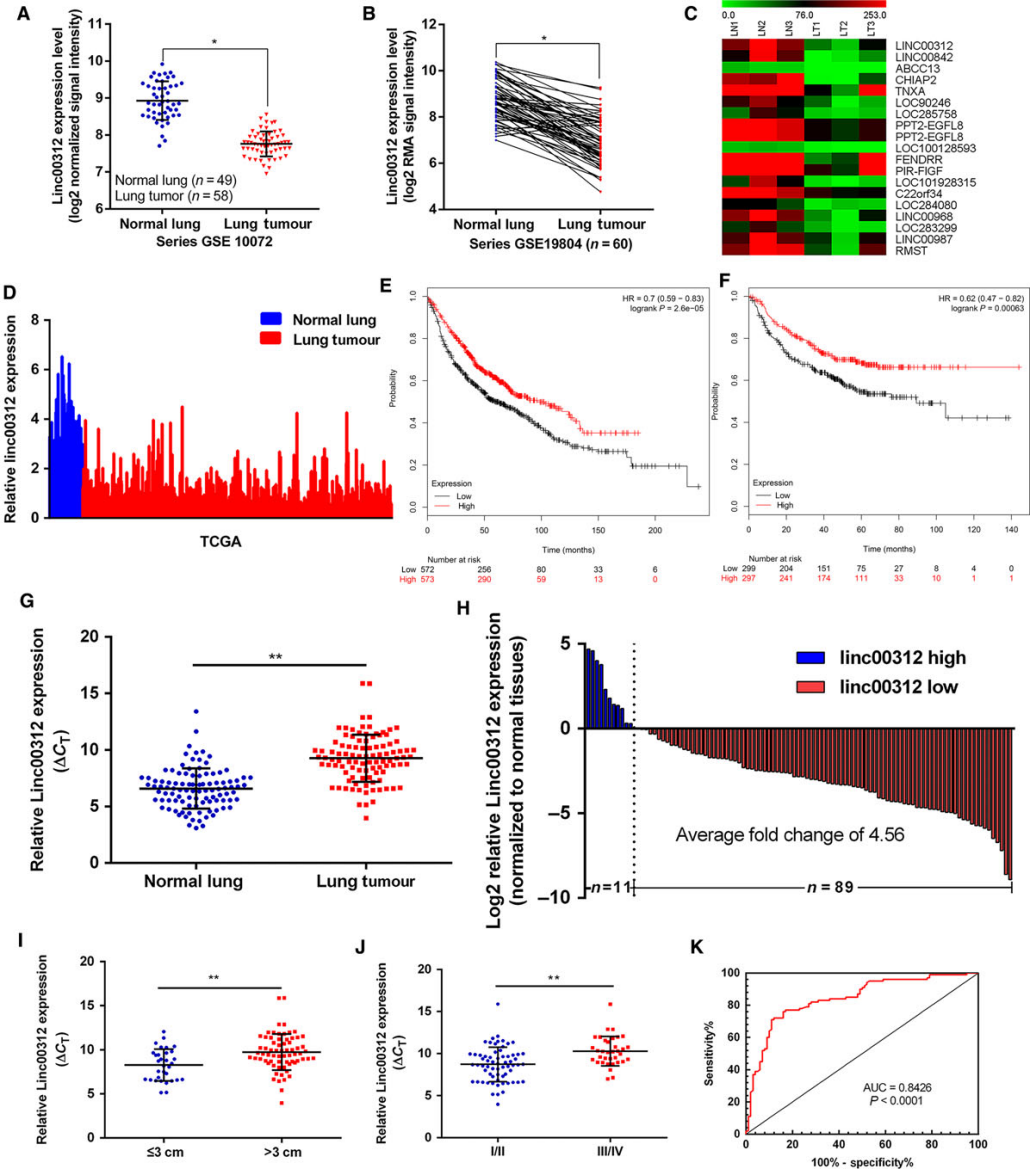
linc00312 was down‐regulated in NSCLC microarray data sets and tissues. **A** and **B**. Relative expression of linc00312 in NSCLC tissues compared with normal tissues was analysed using GEO data sets including series GSE10072 and GSE19804. **C**. A heatmap showed that linc00312 was significantly down‐regulated in GSE66654; green: down‐regulated lncRNAs; red: up‐regulated lncRNAs. **D**. Relative expression of linc00312 in NSCLC tissues compared with normal tissues was analysed using TCGA data. **E** and **F**. Kaplan–Meier analysis (http://kmplot.com/analysis/) of overall survival (**E**) and progression‐free survival (**F**) according to linc00312 expression levels. **G** and **H**. linc00312 expression level in 100 pairs of NSCLC tissues and matched adjacent non‐tumour tissues was examined by qRT‐PCR. linc00312 was obviously down‐regulated in lung cancer tissues (**G**). Expression levels were shown as log2‐fold change to matched non‐tumour tissues (**H**). **I** and **J**. linc00312 expression was lower in tumours with maximum diameter >3 cm and III/IV stages. **K**. Based on the expression level of linc00312, the ROC curve for prediction of NSCLC was analysed using paired adjacent non‐tumorous tissues as a control. The cut‐off value was 7.963 (ΔCT) with sensitivity of 77% and specificity of 83%. The AUC is 0.8426 (95% confidence interval: 0.7873 to 0.8978, *P* < 0.0001). *P < 0.05,**P < 0.01.

The previous study showed that linc00312 was one of the most down‐regulated lncRNAs in NSCLC tumours from the analysis of two GEO data sets (GSE19188 and GSE18842) 18. To identify that linc00312 was involved in NSCLC development and progression, we firstly analysed ncRNAs alterations between NSCLC and non‐tumour tissues in three microarray data sets (GSE10072, GSE19804 and GSE66654) obtained from GEO. We found that linc00312 was down‐regulated in all three data sets (Fig. 1A,B and C). In addition, TCGA lung cancer and normal lung tissues RNA sequencing data also showed that linc00312 expression was down‐regulated in tumour tissues compared with normal tissues (Fig. 1D). Also, linc00312 expression level was associated with smoking status in ADC by analysis of TANRIC data. The expression level of linc00312 was obviously higher in lifelong non‐smoker compared with current smoker and current reformed smoker (Figure S1). Furthermore, the Kaplan–Meier survival analysis showed that patients with lower linc00312 expression levels had a shorter overall survival (OS) (Fig. 1E) and progression‐free survival (PFS) (Fig. 1F) time than those with high expression. These data suggest that linc00312 might be down‐regulated and correlated with poor prognosis in NSCLC.

### Down‐regulation of linc00312 in NSCLC tissues and association with clinicopathological parameters of NSCLC

To validate the analysis finding, we examined and quantified the expression of linc00312 by qRT‐PCR in 100 paired clinical NSCLC tissues and matched non‐tumour tissues. As presented in Figure 1G and H, linc00312 was down‐regulated (4.56‐fold change) in clinical NSCLC tissues (*P* < 0.05). To assess the correlation of linc00312 expression with clinicopathologic characteristics, the expression levels of linc00312 in tumour tissues were categorized as low (*n* = 49, fold change ≥median) or high (*n* = 51, fold change <median) group. Then, we evaluated the correlation of linc00312 expression with patients’ clinicopathological parameters to assess its clinical significance (Table 1). As shown, the linc00312 expression level was significantly lower in larger tumours (maximum diameter >3 cm) or more advanced tumours (*P* < 0.05, Fig. 1I and J). Nevertheless, there was no significant relationship between linc00312 expression and other clinical characteristics (*P* > 0.05, Table 1). We next used paired adjacent non‐tumorous tissues as a control to analyse the prediction values of linc00312 in NSCLC by receiver operating characteristic (ROC) curve. The cut‐off value was 7.963 (ΔCT) with sensitivity at 77% and specificity at 83%, and the AUC is 0.8426(95% confidence interval:0.7873 to 0.8978, *P* < 0.0001) (Fig. 1K). Taken together, the dysregulated linc00312 may serve as a key regulatory factor or biomarker of NSCLC.

**Table 1:** Correlation between linc00312 expression and clinicopathological parameters of NSCLC

Clinicopathological parameters	Number of patients	Linc00312 expression		*P* value
		Low (≤0.139)	High (>0.139)	
All patients	100	49	51	
Gender				
Male	70	34	36	1
Female	30	15	15	
Age				
≤65 y. o.	66	35	31	0.296
>65 y. o.	34	14	20	
Size of tumour				
≤3 cm	33	8	25	0.001**
>3 cm	67	41	26	
Differentiation				
Well/moderate	76	38	38	0.816
Poor	24	11	13	
Lymph node metastasis (pN)				
N0	58	24	34	0.105
N1‐3	42	25	17	
Metastasis (pM)				
M0	96	47	49	1
M1	4	2	2	
p‐TNM stages				
I/II	65	26	39	0.021*
III‐IV	35	23	12	
Position				
left lung	63	32	31	0.682
Right lung	37	17	20	
Histology type				
Adenocarcinoma	57	29	28	0.691
Squamous carcinoma	43	20	23	
Smoking				
Yes	41	22	19	0.542
No	59	27	32	

Chi‐square test. *P < 0.05, **P < 0.001.

### Linc00312 inhibits NSCLC cells proliferation *in vitro*

**Figure 2:**
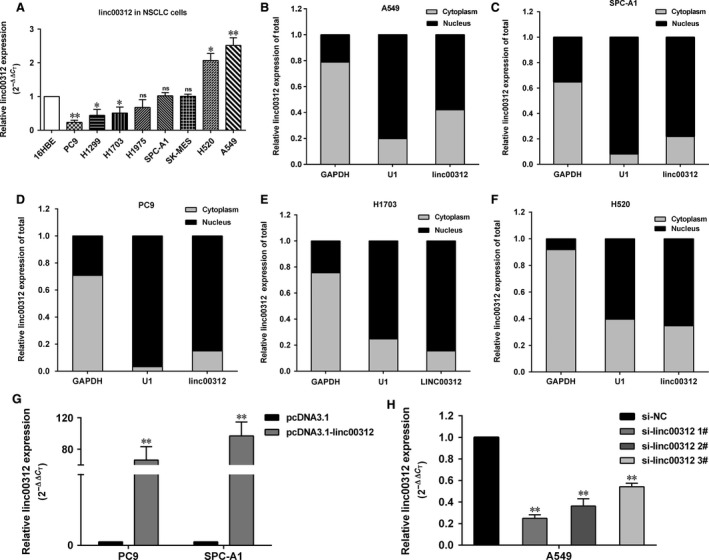
The abundance of linc00312 in NSCLC cells. **A**. Relative expression of linc00312 in NSCLC (PC9, H1299, H1703, H1975, SPC‐A1, SK‐MES, H520 and A549) and human bronchial epithelial cell (16HBE). **B–F**. qRT‐PCR analysis of linc00312 expression levels in different subcellular fractions in A549 (**B**), SPC‐A1 (**C**),PC9 (**D**), H1703 (**E**) and H520 (**F**) cells. GAPDH and small nuclear RNA U1 were utilized as control of cytoplasm and nucleus, respectively. **G**. Relative expression of linc00312 in PC9 and SPC‐A1 cells transfected with pcDNA3.1‐linc00312 vector. **H**. Relative expression of linc00312 in A549 cells transfected with siRNAs. Data are shown as the mean ± S.D.. Based on at least three independent experiments. *P < 0.05, **P < 0.01.

To explore the function of linc00312 in NSCLC, we determined the expression levels in eight NSCLC cells (PC9, H1299, H1703, H1975, SPC‐A1, A549, H520 and SK‐MES) and human bronchial epithelial cell (16HBE). We found that linc00312 expression was obviously lower in four NSCLC cells and higher in A549 cells (Fig. 2A). Furthermore, the localization of linc00312 in cells was determined. Results showed that linc00312 localized in both nucleus and cytosol, but more is in nucleus (Fig. 2B–F).

**Figure 3:**
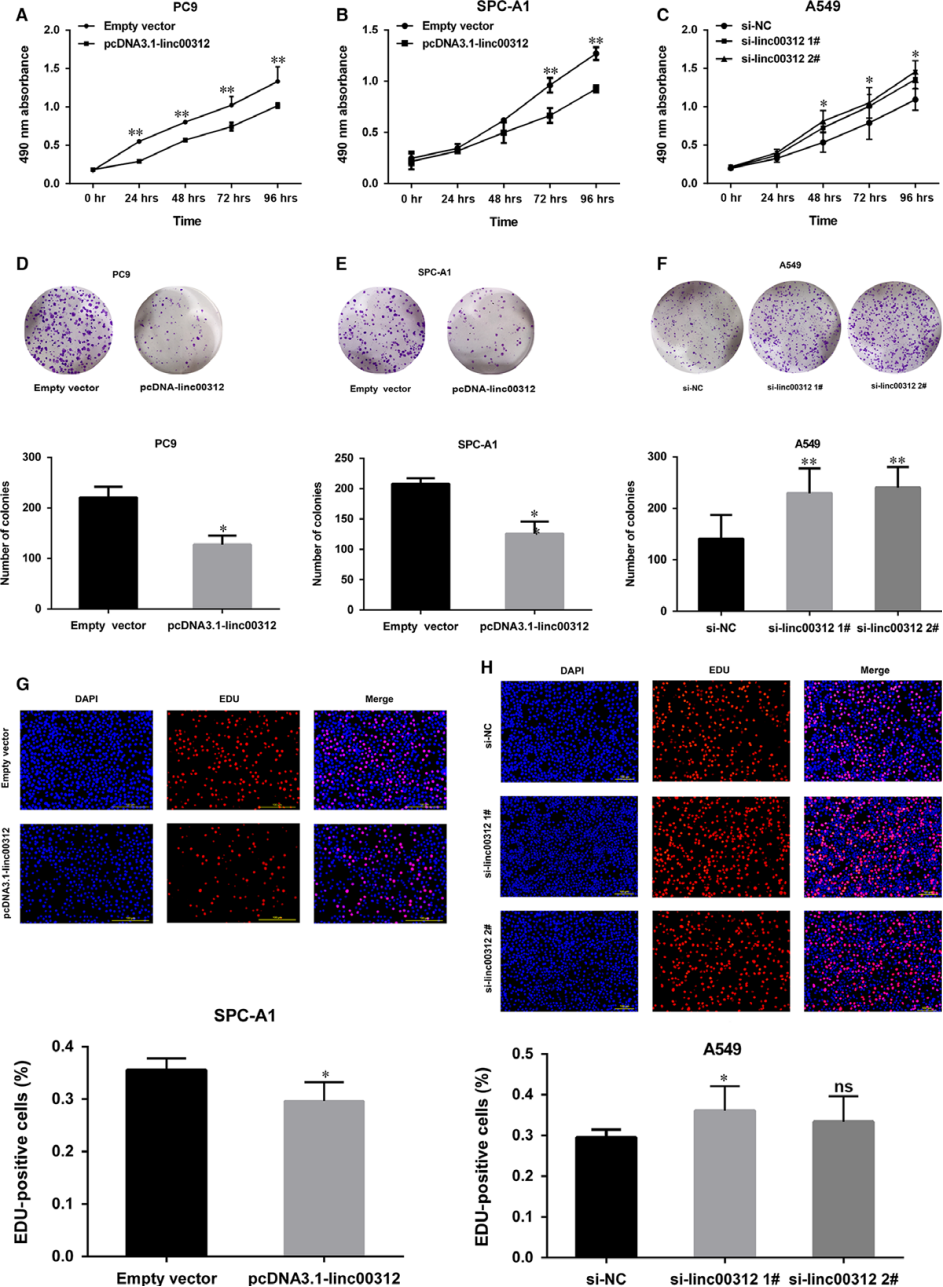
Effects of linc00312 on NSCLC cell proliferation *in vitro*. **A‐C**. MTT assays were performed to determine the cell viability for pcDNA3.1‐linc00312 transfected PC9 and SPC‐A1 cells and si‐linc00312 transfected A549 cells. **D–F**. Colony‐forming assays were used to determine the colon ability of pcDNA3.1‐linc00312 transfected PC9 and SPC‐A1 cells and si‐linc00312 transfected A549 cells. **G** and **H**. EDU staining assays were performed to determine the growth of pcDNA3.1‐linc00312 transfected SPC‐A1 cells and si‐linc00312 transfected A549 cells. Based on at least three independent experiments. *P < 0.05, **P < 0.01.

To further investigate the role of linc00312 in the progression of NSCLC, linc00312 was overexpressed in SPC‐A1 and PC9 with pcDNA3.1‐linc00312 and knocked down in A549 by transfection with siRNAs (Fig. 2G and H). Next, we performed loss or gain of function assays. The MTT assays showed that the cell viability was decreased in linc00312 overexpressed PC9 and SPC‐A1 cells, while increased in si‐linc00312‐treated A549 cells (Fig. 3A–C). Consistently, the colony formation assays showed that linc00312 overexpression resulted in impairment of PC9 and SPC‐A1 colon formation ability, while linc00312 knock‐down increased A549 colon formation ability (Fig. 3D–F). In addition, the EDU staining assays showed the same results (Fig. 3G and H). Collectively, linc00312 may play an important role in NSCLC cell proliferation.

**Figure 4:**
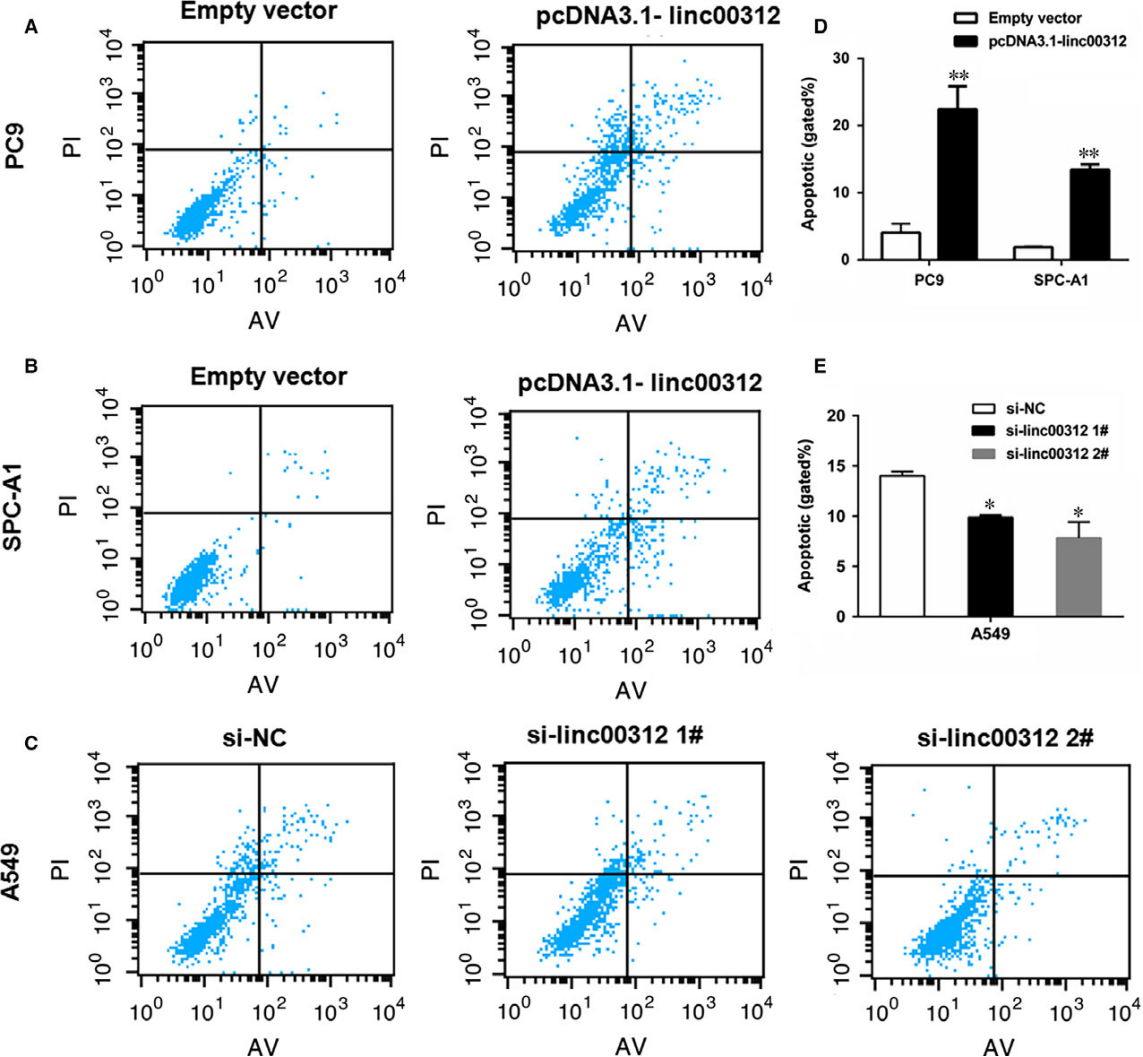
The effect of linc00312 on NSCLC cell apoptosis. **A–C**. Flow cytometry cell apoptosis assays were used to analyse the cell apoptotic in pcDNA3.1‐linc00312 transfected PC9 and SPC‐A1 cells and si‐linc00312 transfected A549 cells. **D**. Statistical analysis of the cell apoptosis in PC9 and SPC‐A1 cells. **E**. Statistical analysis of the cell apoptosis in A549 cells. Based on at least three independent experiments. *P < 0.05, **P < 0.01.

To determine whether linc00312 promoting NSCLC cell proliferation was influenced by apoptosis, we performed flow cytometric analysis. The results showed that the fraction of apoptotic cells was significantly increased among the linc00312 overexpressed cells, while was decreased in si‐linc00312‐treated cells (Fig. 4), confirming that linc00312 was involved in the NSCLC cell apoptosis .

### Deacetylation and methylation may not be involved in the down‐regulation of linc00312

**Figure 5:**
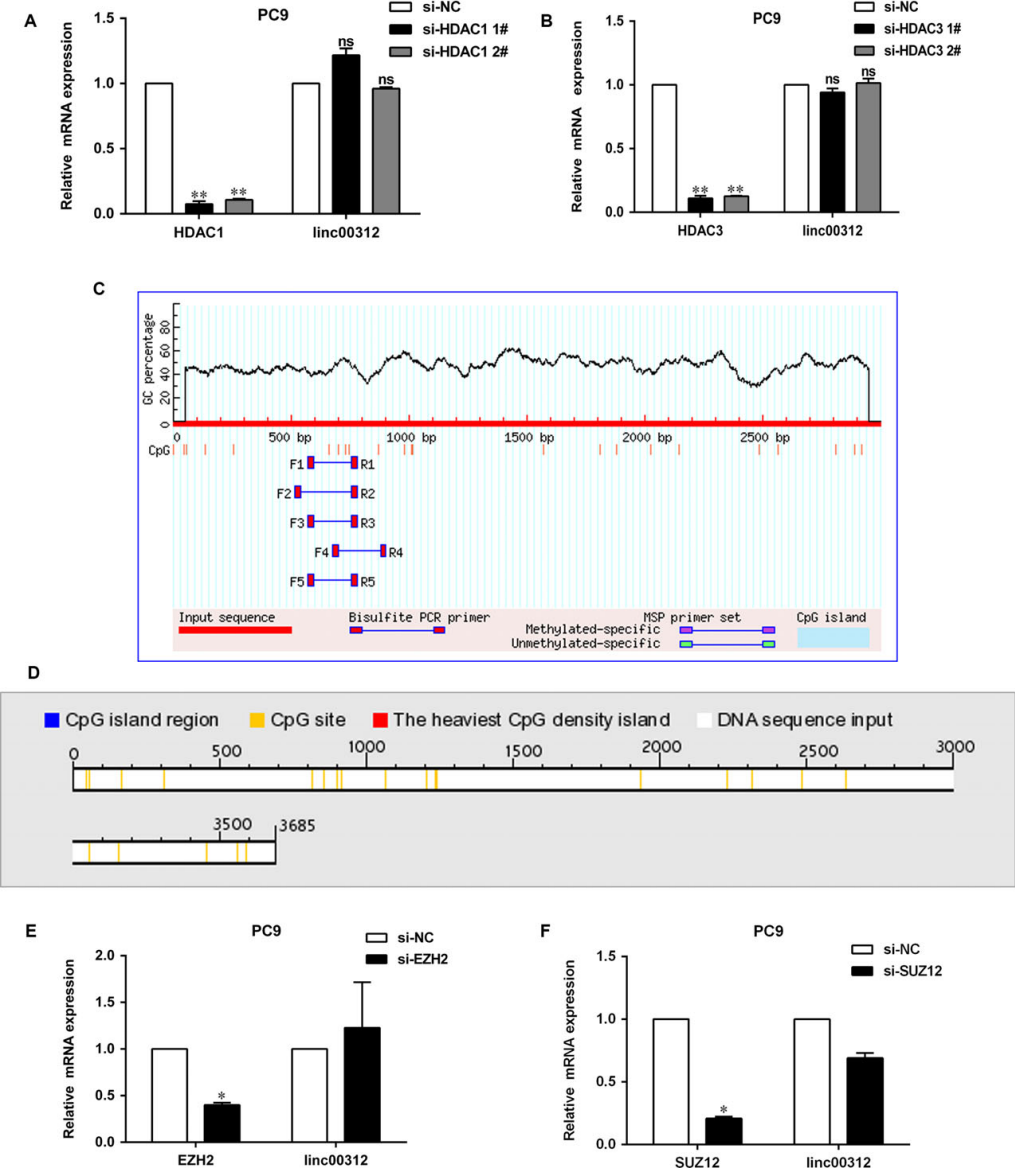
Deacetylation and methylation may not involve in the down‐regulation of linc00312. **A** and **B**. qRT‐PCR analysis of linc00312 expression after knocked down HDAC1 and HDAC3 in PC9 cell. **C** and **D**. CpG islands were analysed by the online databases. **E** and **F**. qRT‐PCR analysis of linc00312 expression after knocked down EZH2 and SUZ12 in PC9 cell. All experiments were performed in triplicates. *P < 0.05, **P < 0.01.

Linc00312 played a vital role in the proliferation of NSCLC; therefore, we set out to determine the regulators of linc00312. Deacetylation and methylation of chromatin could silence the expression of genes. Histone deacetylases (HDAC) are a class of enzymes that remove acetyl groups from a histone, allowing the histones to wrap the DNA more tightly. The expression of linc00312 was not increased after inhibiting HDAC1 or HDAC3 in PC9, indicating that deacetylation may not be involved in the down‐regulation of linc00312 (Fig. 5A and B). Next, we sought to find out whether methylation could regulate the expression of linc00312. There were no CpG islands in the promoter of linc00312 by the analysis of the promoter sequence of linc00312 using the online software (http://www.urogene.org/cgi-bin/methprimer/methprimer.cgi) and (http://dbcat.cgm.ntu.edu.tw/) (Fig. 5C and D). Then, we explored whether histones methylation influences linc00312. The polycomb repressive complex 2 (PRC2) catalyses trimethylation of lysine 27 of histone H3 (H3K27me3) to repress gene transcription. SUZ12 (zinc finger) and enhancer of zeste homolog 2 (EZH2, SET domain with histone methyltransferase activity) knocking down (using siRNA) could not induce linc00312 in PC9 cell (Fig. 5E and F). Together, our results preliminarily indicated that deacetylation and methylation may not be involved in the down‐regulation of linc00312.

### Transcription factor HOXA5 regulated the expression of linc00312

**Figure 6:**
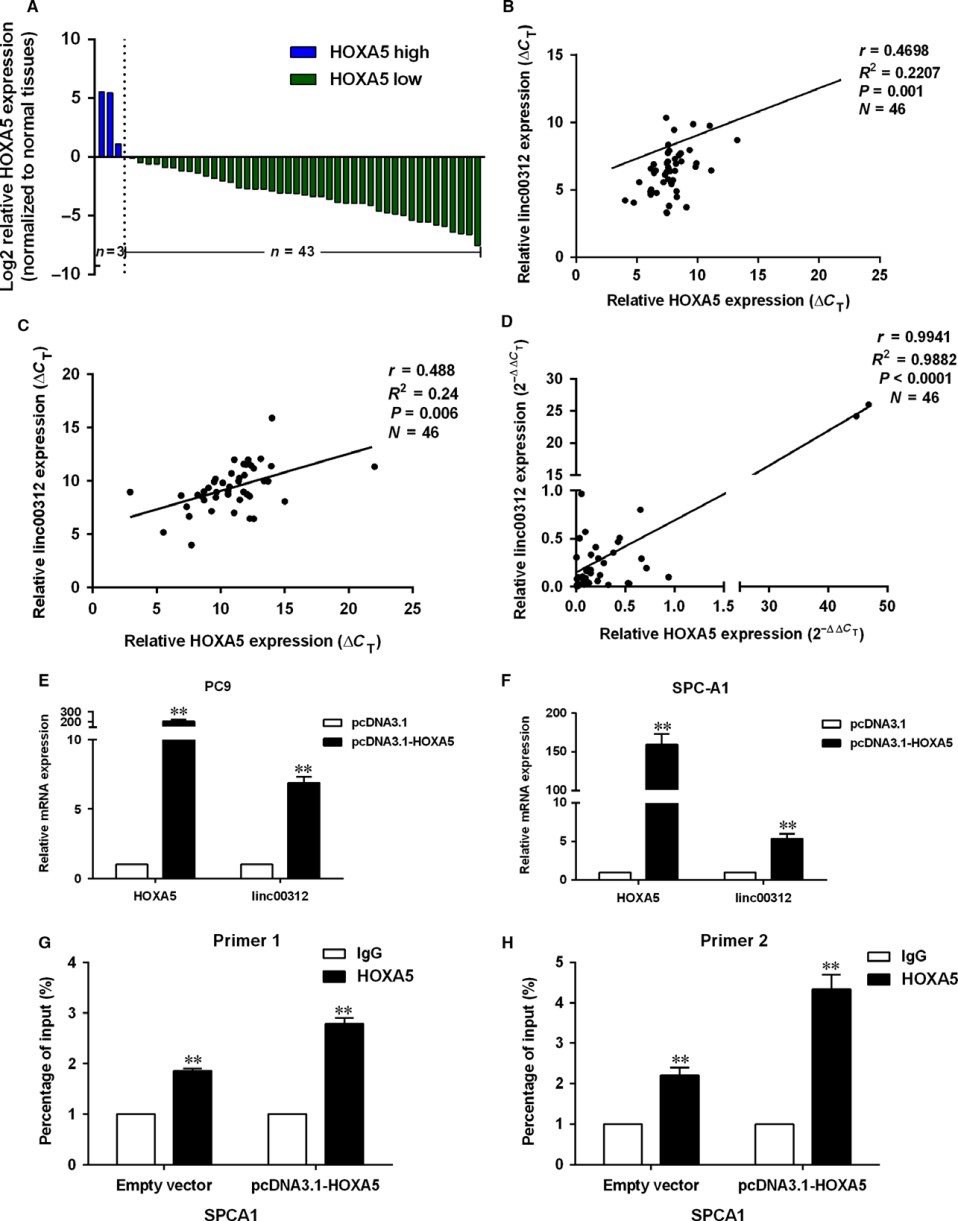
HOXA5 regulated the expression of linc00312. **A**. HOXA5 mRNA level in 46 pairs of NSCLC tissues and matched non‐tumour tissues was examined by qRT‐PCR. **B** and **C**. Correlation of linc00312 and HOXA5 mRNA expression levels in 46 normal lung tissues (**B**) and lung cancer tissues (**C**) (ΔCT value). **D**. Correlation of linc00312 and HOXA5 mRNA expression levels in 46 paired NSCLC and adjacent normal lung tissues (2^−ΔΔCT^ value). **E** and **F**. qRT‐PCR analysis of linc00312 expression after increased HOXA5 expression in PC9 and SPC‐A1 cell. **G** and **H**. ChIP‐qPCR analysis of HOXA5 occupancy in the promoter of linc00312. All experiments were performed in triplicates. **P < 0.01.

Transcription factors (TFs) have a central role in the regulation of gene expression. To further explore the regulator of linc00312, we analysed the TFs which might bind with the promoters of linc00312 by the JARSPAR databases (Supplement Table S3). The results showed that HOXA5, which was reported down‐regulated in NSCLC, is included in the TFs. To determine whether HOXA5 could influence the expression of linc00312, the expression level of HOXA5 mRNA in 46 of 100 paired clinical NSCLC tissues and adjacent normal tissues was examined. Results indicated that HOXA5 mRNA was down‐regulated in NSCLC tissues, which is consistent with the previous study (Fig. 6A). The HOXA5 mRNA expression level was positively correlated with linc00312 in these samples (Fig. 6B–D). In addition, increasing HOXA5 expression resulted in the up‐regulation of linc00312 (Fig. 6E and F). Furthermore, ChIP experiments results showed that HOXA5 could bind with the promoters of linc00312 (Fig. 6G and H). All these results indicated that HOXA5 may serve as a regulator of linc000312.

### Linc00312 overexpression suppressed NSCLC tumour growth *in vivo*

**Figure 7:**
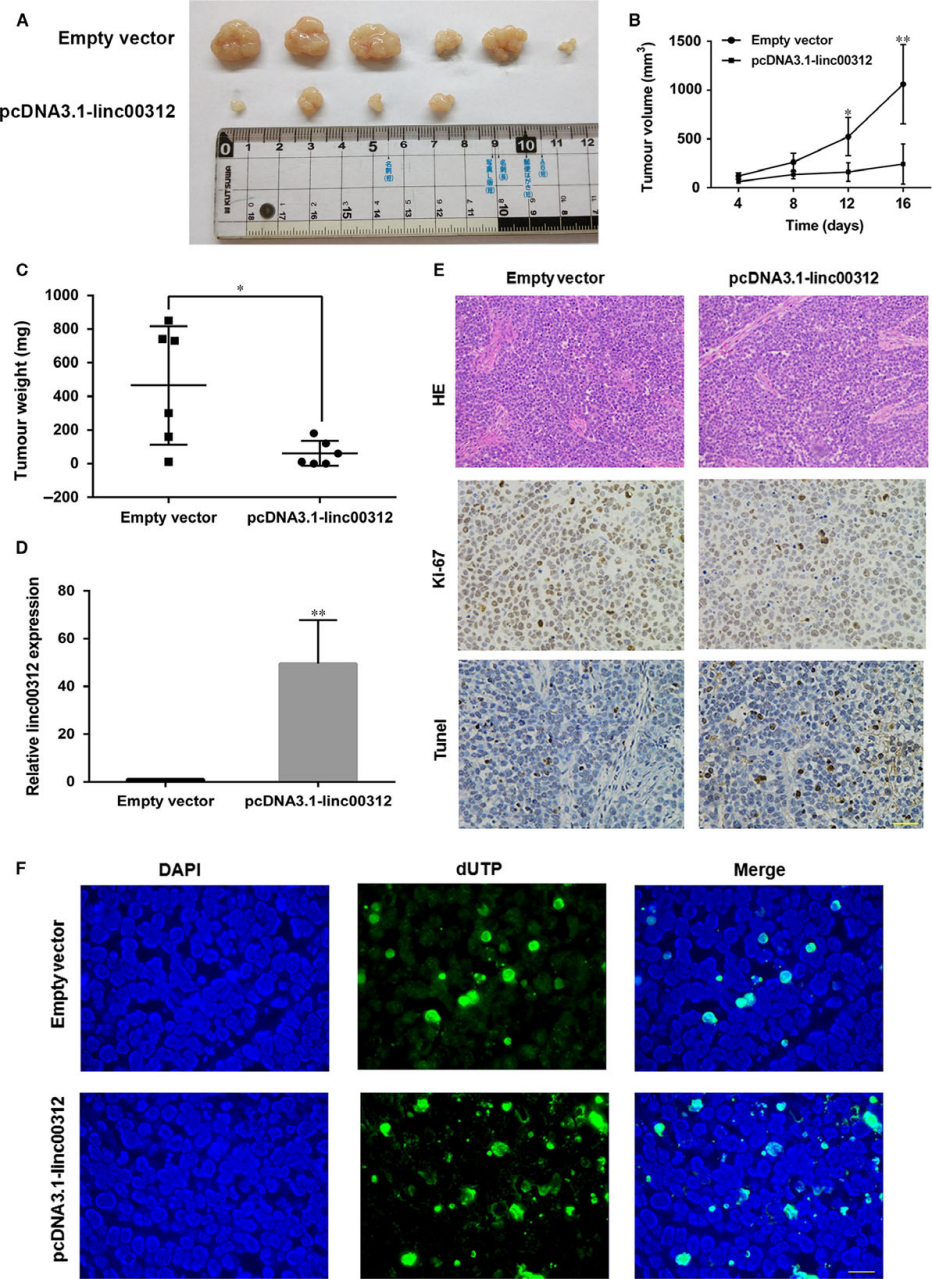
Linc00312 overexpression suppressed NSCLC tumour growth *in vivo*. **A**. Representative photograph of tumour formation: pcDNA3.1 and pcDNA3.1‐linc00312 cells were subcutaneously incubated in nude mice up to 16 days. **B** and **C**. Statistical analysis of tumour volumes (**B**) and tumour weight changes (**C**) *in vivo*. **D**. qRT‐PCR analysis of linc00312 expression in tumour tissues after linc00312 overexpressing *in vivo*. **E**. ICH of HE, ki‐67 and tunnel of the tumour tissues *in vivo*. Scale bar represents 100 μm. **F**. Tunel staining assays were conducted to analyse the cell apoptosis of tumour tissues after linc00312 overexpressing *in vivo*. Scale bar represents 50 μm. Data are presented as mean ± S.E.*P < 0.05, **P < 0.01.

To further investigate whether linc00312 expression could affect tumour growth *in vivo,* SPC‐A1 cells stably transfected with empty vector or pcDNA3.1‐linc00312 were inoculated into male nude mice. Sixteen days after injection, the tumour size in the pcDNA3.1‐linc00312 group was significantly smaller compared with the control group (Fig. 7A–B). The tumour weight of pcDNA3.1‐linc00312 group was also significantly lower than that in the control group (Fig. 7C). Next, qRT‐PCR assays determined that linc00312 expression levels were down‐regulated in tumour tissues collected from pcDNA3.1‐linc00312 group compared with control group (Fig. 7D). Moreover, immunohistochemistry (IHC) analysis confirmed that the tumours formed from pcDNA3.1‐linc00312 transfecting cells displayed lower Ki‐67 staining and higher tunnel staining than those formed from the control cells (Fig. 7E and F). Together, our results indicated that increasing linc00312 expression could suppress NSCLC tumour growth *in vivo*.

### Linc00312 was down‐regulated in the plasma of NSCLC patients

**Figure 8:**
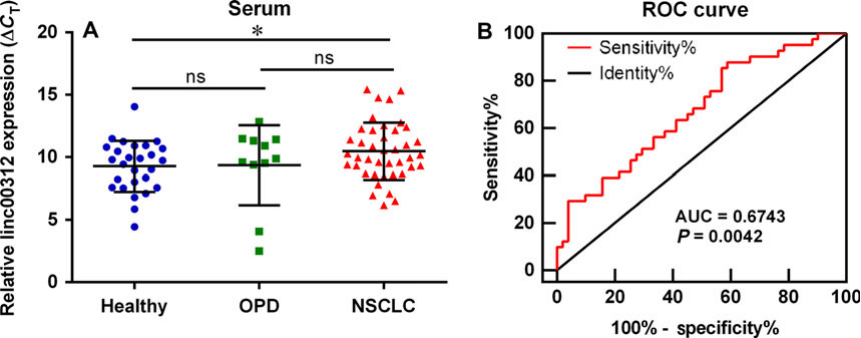
linc00312 was down‐regulated in the plasma of NSCLC patients. **A**.Relative expression of linc00312 in the plasma of 41 NSCLC patients, 11 other pulmonary diseases (OPD) patients and 27 healthy volunteers. *P < 0.05. ns = not significant. **B**. Based on the expression level of linc00312, the ROC curve for prediction of NSCLC was analysed using healthy volunteers and OPD as a control. The cut‐off value was 8.418 (ΔCT) with sensitivity at 87.8% and specificity at 41.18% and the AUC is 0.6743 (95% confidence interval:0.5648 to 0.7838, *P* = 0.0042).

A major factor of the poor prognosis of NSCLC is the difficulties in detecting in the early stage, resulting in delayed disease therapies. Therefore, a better understanding of the biomarkers of NSCLC is needed to improve early detection and disease intervention. To this end, we examined the expression level of linc00312 in the plasma of NSCLC patients, other pulmonary diseases (OPD) patients and healthy volunteers. The results showed that linc00312 was obviously down‐regulated in the plasma of NSCLC patients compared with that of healthy control. Surprisingly, there was no significant statistic difference between the expression level of linc00312 in the plasma of the OPD patients and healthy volunteers, which indicate the perfect specificity of linc00312 as a biomarker of NSCLC (Fig. 8A). Thus, we analysed the prediction values of linc00312 in the plasma of NSCLC patients by ROC curve. The cut‐off value was 8.418 (ΔCT) with sensitivity at 87.8% and specificity at 41.18%, and the AUC is 0.6743 (95% confidence interval:0.5648 to 0.7838, *P* = 0.0042) (Fig. 8B). Collectively, the down‐regulated linc00312 may serve as a biomarker of detection of NSCLC.

## Discussion

As shown in Supplement Table S1, a handful of lung cancer‐related lncRNAs have been characterized for their biological functions and clinical implications. Even though emerging roles of lncRNAs in NSCLC have been highlighted 21, 22, 23, 24, lung cancer‐related lncRNAs are still an emerging field compared to the vast majority of lncRNAs in the human genome and further characterization of these lung cancer‐associated lncRNAs will provide a better understanding of their potential roles as biomarker or therapeutic targets. In the current study, we discovered that the lincRNA00312 was down‐regulated in NSCLC tumours compared to the adjacent normal lung tissues, which was consistent with the previous study 18 and confirmed the analysis of the GEO and TCGA data sets. Specifically, the lower expression of linc00312 was significantly lower in larger and later stages tumours and was correlated with poor prognosis. A ROC analysis was performed to verify the ability of the linc00312 expression to differentiate between non‐cancerous and NSCLC patients. Results showed that the AUC of linc00312 could be useful for distinguishing non‐cancerous patients from NSCLC patients, consistent with previous study of NPC 16. These results indicated that linc00312 may play an important role in NSCLC carcinogenesis. Thus, exploring biological significance and the molecular mechanisms of linc00312 in NSCLC carcinogenesis is necessary. To this end, loss and gain function of linc00312 in NSCLC cells were used. Overexpression of linc00312 impaired NSCLC cells growth and induced cell apoptosis *in vitro* and *in vivo*. These results uncovered the tumour suppressor role of linc00312 in NSCLC.

Epigenetic events, including deacetylation and methylation of chromatin, could modulate the expression of genes. Loss of expression of tumour suppressor genes could be regulated through reversible deacetylation of lysine residues. Histone deacetylases (HDACs) promote the deacetylation of lysine residues on histones and play important roles in various diseases 25. HDAC1 and HDAC3 were reported to modulate the expression of several lncRNAs 26, 27, 28.Accordingly, we determined the effect of HDAC1 or HDAC3 on linc00312 and found that inhibiting HDAC1 or HDAC3 could not up‐regulate the expression of linc00312. DNA methylation of CpG islands at promoter sites is believed to contribute to tumorigenesis through transcriptional silencing of tumour suppressor genes. However, there were no CpG islands in the promoter of linc00312. Histone modifications are closely associated with the function of polycomb group proteins. An important player in this silencing process is the polycomb repressive complex 2 (PRC2) 29. EZH2 is the H3K27 methyltransferase catalytic subunit of PRC2, and SUZ12 is zinc finger of it. However, the expression of linc00312 was not increased after inhibiting EZH2 or SUZ12. We tentatively put forward that histone deacetylation and methylation may not influence the expression of linc00312. However, we have to point out our results need more supports. Notwithstanding its limitation, this study does suggest detecting the changes in linc00312 with deacetylation and methylation inhibitors.

HOXA5, as tumour suppressor, is involved in many cancers progression, including lung cancer 19, 20, 30, 31, 32, 33, 34. HOXA5 inhibited NSCLC cell proliferation and invasion by interacting with p21 in *vitro and in vivo* 20. Also, miR‐1271 and miR‐196a promoted NSCLC cell proliferation and invasion *via* the down‐regulation of HOXA5 31, 33. Here, we confirmed that HOXA5 was significantly down‐regulated in paired NSCLC tumours. Furthermore, HOXA5 could bind with the promoter of linc00312 and influence its expression. However, the HOXA5‐binding sites at the regions in the linc00312 promoter are not determined.

Liquid biopsies are technological advances as a recent breakthrough in the precision medicine. Liquid biopsies in non‐invasive can detect cancer signs from a blood sample, including circulating tumour cells, circulating tumour DNA, exosomes *et al*. 35. Prior studies have noted the importance of detecting circulating miRNAs in the plasma of gastric cancer 36, oesophageal squamous cell carcinoma 37, hepatocellular carcinoma 38 and lung cancer 39. Nevertheless, little is known about circulating lncRNAs in NSCLC. We previously found GAS5 was detectable and stable in the plasma of NSCLC patients and GAS5 expression levels could be used to distinguish NSCLC patients from control patients with an area under the ROC of 0.832 40. Here, we found that linc00312 was significantly lower in the plasma of NSCLC compared with that in the healthy volunteers or OPD patients. It is worth mentioning that there was no significant statistic difference between the expression level of linc00312 in the plasma of the OPD patients and healthy volunteers. Unfortunately, the number of OPD patients is only 11, which may influence the diagnosis accuracy analysis of linc00312 by ROC, and further investigation with a larger pool of patients is warranted. Also, further studies about stability of linc00312 in blood are needed. Despite its preliminary character, this study does suggest analysing diagnosis accuracy of combination of linc00312 and other biomarkers of NSCLC.

In summary, this study identified linc00312 as a novel potential tumour suppressor in NSCLC and played its role partly by inhibiting tumour growth and inducing apoptosis *in vitro* and *in vivo*. HOXA5 may regulate the expression of linc00312. The lower expression of linc00312 in plasma indicated that linc00312 might serve as a tumour biomarker for NSCLC diagnosis. Studies are needed to explore the targets and the mechanism that underlie regulatory behaviours of linc00312. We used RIP‐qPCR detected that linc00312 could not bind with EZH2, SUZ12, LSD1 and DNMT1 (data not shown). In addition, bioinformatics analysis showed it may interact with RNA binding protein FUS (analysis of starBase data) and further experiments are needed to verify this interacting. However, other possible biological behaviours of linc00312 were not investigated in this study, which needs further investigation.

## Conflict of interest

The authors confirm that there are no conflict of interests.

## Supporting information


**Figure S1** Linc00312 expression level was associated with smoking status in ADC by analysis of TANRIC data. The expression level of linc00312 was obviously higher in lifelong non‐smoker compared with current smoker and current reformed smoker. *P* = 0.048037.

**Table S1** Lung cancer‐related lncRNAs.

**Table S2** Primers used for qRT‐PCR or ChIP and siRNAs oligonucleotides.

**Table S3** TFs which might bind with the promoters of linc00312 by the JARSPAR databases (http://jaspar.binf.ku.dk/cgi-bin/jaspar_db.pl)
