# Supplementary figures and images for: Long non‐coding RNA 00312 regulated by HOXA5 inhibits tumour proliferation and promotes apoptosis in Non‐small cell lung cancer
Source: J Cell Mol Med. 2017 Mar 24;21(9):2184–98. doi: 10.1111/jcmm.13142 (PMC5571553; doi:10.1111/jcmm.13142)

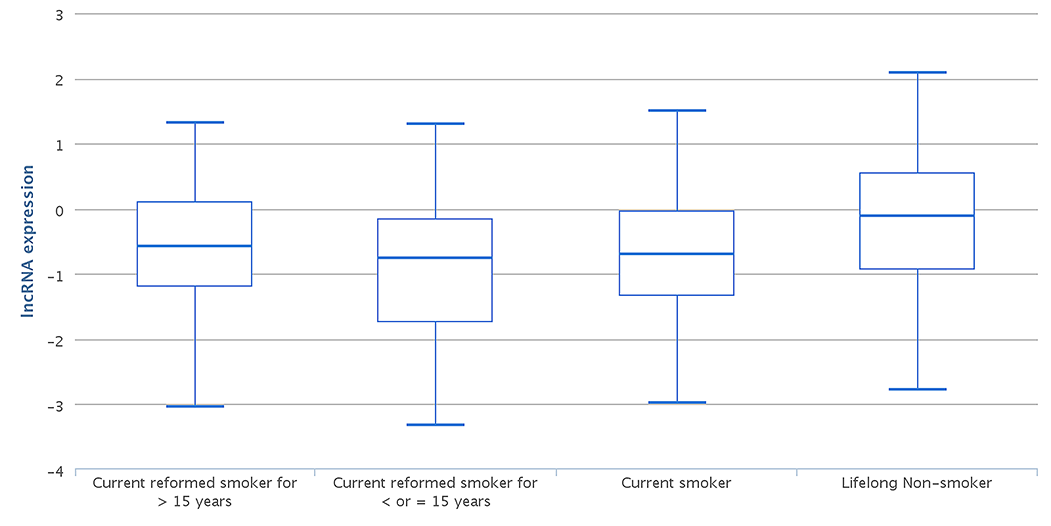

Supplement: Supplementary file 1 — Figure S1 Linc00312 expression level was associated with smoking status in ADC by analysis of TANRIC data. The expression level of linc00312 was obviously higher in lifelong non‐smoker compared with current smoker and current reformed smoker. P = 0.048037. [file JCMM-21-2184-s001.tif]
